# Dehydration independently predicts prolonged hospital stay following aseptic revision total shoulder arthroplasty

**DOI:** 10.1007/s00590-025-04323-3

**Published:** 2025-05-20

**Authors:** Steven H. Liu, Allen Bramian, Rachel A. Loyst, Edward D. Wang

**Affiliations:** 1 University of Southern California, Los Angeles, United States; 2 Stony Brook Medicine, Stony Brook, United States

**Keywords:** Revision total shoulder arthroplasty, Total shoulder arthroplasty, Dehydration, Blood urea nitrogen, Creatinine, Length of stay, Complications

## Abstract

**Background:**

This study investigates the association between preoperative dehydration and 30-day postoperative complications following aseptic revision total shoulder arthroplasty (TSA).

**Methods:**

The American College of Surgeons National Surgical Quality Improvement Program database was queried for all patients who underwent aseptic revision TSA from 2015 to 2022. The study population was divided into two groups based on preoperative hydration status: normal (blood urea nitrogen (BUN)/creatinine (Cr) < 25) and dehydration (BUN/Cr ≥ 25). Logistic regression analysis was conducted to investigate the relationship between preoperative dehydration and postoperative complications.

**Results:**

Compared to normal hydration, dehydration was associated with a significantly greater likelihood of experiencing any complication (*P* = 0.004), nonhome discharge (*P* = 0.002), and length of stay (LOS) > 2 days (*P* < 0.001). After controlling for potential confounding variables with multivariate analysis, dehydration was independently significantly associated with LOS > 2 days (odds ratio 1.50, 95% confidence interval 1.05–2.14; *P* = 0.028).

**Conclusion:**

Preoperative dehydration status is associated with a greater rate of early postoperative complications and is an independent predictor of LOS exceeding two days following aseptic revision TSA. A better understanding of dehydration as a risk factor for postoperative complications may help surgeons better select surgical candidates and improve surgical outcomes in the setting of aseptic revision TSA.

**Level of evidence:**

Level III; Retrospective Cohort Comparison; Prognosis Study.

## Introduction

Total shoulder arthroplasty (TSA) utilization continues to rise, with a notable increase of 103.7% between the years of 2011 and 2017 [1]. Multiple studies have noted a concurrent rise in revision TSA, which has been associated with elevated postoperative complication rates [2–5]. As such, identifying modifiable preoperative risk factors for postoperative complications following revision TSA may enhance patient risk assessment and management.

Dehydration, defined by a blood urea nitrogen (BUN) to creatinine (Cr) ratio ≥ 25, can lead to symptoms ranging from fatigue to hemodynamic instability. Studies investigating preoperative dehydration as a risk factor for postoperative complications following orthopedic surgery are limited. However, following total knee, ankle, and shoulder arthroplasty, an association between dehydration and increased length of stay (LOS) has been revealed [6–8]. Additionally, dehydrated patients undergoing orthopedic surgery were found to have a significantly higher incidence of postoperative complications in both the respiratory and gastrointestinal systems, as well as an increased risk of mortality, compared to those with normal hydration [9]. These findings warrant investigation into the role of dehydration in other orthopedic procedures, particularly procedures such as revision TSA with higher morbidity. Investigation into dehydration as a preoperative risk factor may provide clinical utility in managing preoperative hydration status.

This study aims to determine the relationship between preoperative dehydration and 30-day postoperative complications following revision TSA. We hypothesized that dehydration would be associated with higher rates of 30-day postoperative complications following aseptic revision TSA.

## Materials and methods

We queried the American College of Surgeons National Surgical Quality Improvement Program (ACS-NSQIP) database for all patients who underwent revision TSA from 2015 to 2022. This study was exempt from approval by our University’s Institutional Review Board as the NSQIP database is fully deidentified. Data in the NSQIP database are gathered from over 600 hospitals in the United States by trained surgical clinical reviewers. The data are periodically reviewed to maintain high reliability.

**Fig. 1:**
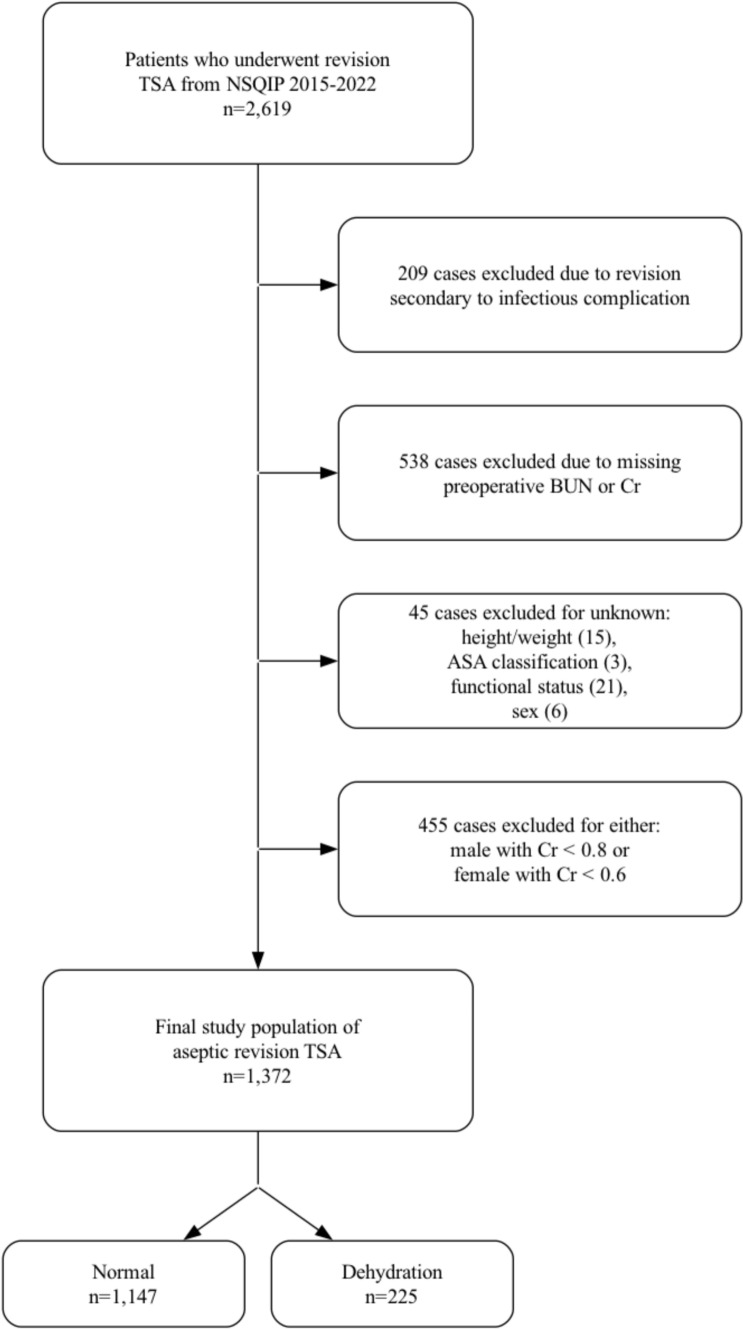
Case selection schematic. *TSA* total shoulder arthroplasty, *NSQIP* National Surgical Quality Improvement Program, *ASA* American Society of Anesthesiologists, *BUN* blood urea nitrogen, *Cr* creatinine

The *Current Procedural Terminology* (CPT) codes 23473 and 23474 were used to identify 2619 patients who underwent revision TSA from 2015 to 2022 (Fig. 1). The NSQIP database inherently excludes all cases for patients younger than 18 years of age and all cases with primary admission related to trauma. Initially, 209 revision TSA cases were excluded due to revision for an infectious etiology. Only aseptic revision TSA cases were included as the NSQIP database lacks details regarding the chronicity of infection (acute vs. chronic), which may significantly impact outcomes. Next, 538 cases were excluded due to either missing BUN or Cr measurements. Additionally, 45 cases were excluded for unknown height/weight data, the American Society of Anesthesiologists (ASA) classification, functional health status, or sex. Furthermore, 455 cases were excluded because patients were either (1) female with Cr < 0.6 or (2) male with Cr < 0.8. In short, Cr values below these thresholds were excluded because they are associated with malnutrition, advanced liver disease, and chronic kidney disease [6, 10]. Including these patients could introduce confounding, as their elevated BUN/Cr ratios may reflect underlying comorbidities rather than true dehydration. The final patient population included in the study after exclusion criteria was 1372, which was then separated into two cohorts: normal (BUN/Cr < 25) with 1147 patients and dehydration (BUN/Cr ≥ 25) with 225 patients. Dehydration was defined as a blood urea nitrogen-to-creatinine (BUN/Cr) ratio ≥ 25, consistent with definitions used in previous orthopedic studies [6–8].

An a priori power analysis was conducted using Python version 3.8 with the Statsmodels Python package to determine the minimum sample size needed to test the study hypothesis. The results of the power analysis indicated the required minimum sample size required to achieve 80% power to detect a medium effect size, at a significance of alpha = 0.05, was *n* = 63 samples per group for a logistic regression analysis. Therefore, the obtained sample sizes of *n* = 1147 and 225, respectively, is adequate to test the study hypothesis.

Variables collected in this study included patient demographics, comorbidities, surgical characteristics, and 30-day postoperative complication data. Patient demographics included sex, age, body mass index (BMI), functional status, ASA classification, smoking status, and preoperative steroid use. Functional status was categorized as “independent” if the patient required no assistance from another individual for any activities of daily living, and as “dependent” if the patient required assistance for some or all such activities. Preoperative steroid use was defined as the routine administration of oral or parenteral corticosteroids for a chronic medical condition within 30 days preceding the surgical procedure. Preoperative comorbidities included congestive heart failure (CHF), diabetes mellitus, hypertension, severe chronic obstructive pulmonary disease (COPD), bleeding disorders, disseminated cancer, and total operation time. Thirty-day complications included the following: any complication, sepsis, septic shock, pneumonia, unplanned reintubation, urinary tract infection (UTI), cardiac arrest or myocardial infarction (MI), stroke, blood transfusions, deep vein thrombosis (DVT), pulmonary embolism (PE), on ventilator > 48 h, surgical space infection (SSI), wound dehiscence, acute renal failure, *Clostridioides difficile*** (***C. diff*) infection, nonhome discharge, readmission, unplanned reoperation, periprosthetic fracture, length of stay (LOS) > 2 days, and mortality. “Any complication” refers to the occurrence of one or more postoperative complications examined in this study.

Statistical analyses were performed using Python version 3.8 with the Statsmodels Python package. Bivariate logistic regression was used to compare patient demographics and comorbidities between the two groups. Multivariate logistic regression, adjusted for all patient demographics and comorbidities significantly associated with dehydration, was used to identify significant independent associations between dehydration and postoperative complications. Odds ratios (ORs) were reported with 95% confidence intervals (CIs). The level of statistical significance was set at *P* < 0.05.

## Results

This study cohort consisted of 1372 patients, 1147 (83.6%) with normal hydration and 225 (16.4%) with dehydration. Of this cohort, 509 (37.1%) were female and 863 (62.9%) were male. Ten (0.01%) were age 18–39 years, 207 (15.1%) were age 40–59 years, 996 (72.6%) were age 60–79 years, and 159 (11.6%) were age ≥ 80 years. One (0.1%) had BMI < 18.5, 199 (14.5%) had BMI 18.5–24.9, 427 (31.1%) had BMI 25–29.9, and 745 (54.3%) had BMI ≥ 30. Forty three (3.1%) were of dependent functional status and 1329 (96.9%) were of independent functional status. Four hundred fifty-nine (33.5%) were ASA classification ≤ 2 and 913 (66.5%) were ASA classification ≥ 3.

Compared to normal hydration, dehydration was significantly associated with female sex (*P* < 0.001), age ≥ 60 years (*P* < 0.001), ASA classification ≥ 3 (*P* = 0.028), and comorbid hypertension (*P* = 0.045) and COPD (*P* = 0.035) (Table 1).

**Table 1:** Demographics and comorbidities of patients with normal hydration and dehydration

Characteristics	Normal (BUN/Cr < 25)	Dehydration (BUN/Cr ≥ 25)	
	Number (%)	Number (%)	*P* value
Overall	1147 (100.0)	225 (100.0)	
Sex			** < 0.001**
Female	395 (34.4)	114 (50.7)	
Male	752 (65.6)	111 (49.3)	
Age			** < 0.001**
18–39	9 (0.8)	1 (0.4)	
40–59	189 (16.5)	18 (8.0)	
60–79	832 (72.5)	164 (72.9)	
≥ 80	117 (10.2)	42 (18.7)	
BMI (kg/m^2^)			0.263
< 18.5	1 (0.1)	0 (0.0)	
18.5–24.9	162 (14.1)	37 (16.4)	
25–29.9	354 (30.9)	73 (32.4)	
≥ 30	630 (54.9)	115 (51.1)	
Functional status prior to surgery			0.394
Dependent	38 (3.3)	5 (2.2)	
Independent	1109 (96.7)	220 (97.8)	
ASA classification			**0.028**
≤ 2	398 (34.7)	61 (27.1)	
≥ 3	749 (65.3)	164 (72.9)	
Smoker			0.068
No	1018 (88.8)	209 (92.9)	
Yes	129 (11.2)	16 (7.1)	
Steroid use			0.784
No	1081 (94.2)	211 (93.8)	
Yes	66 (5.8)	14 (6.2)	
Comorbidities			
CHF	22 (1.9)	8 (3.6)	0.131
Diabetes	0 (0.0)	0 (0.0)	0.394
Hypertension	806 (70.3)	173 (76.9)	**0.045**
COPD	76 (6.6)	24 (10.7)	**0.035**
Bleeding disorder	43 (3.7)	10 (4.4)	0.621
Disseminated cancer	3 (0.3)	0 (0.0)	0.999
Total operation time (min)			0.566
0–79	267 (23.3)	51 (22.7)	
80–128	451 (39.3)	84 (37.3)	
≥ 129	429 (37.4)	90 (40.0)	

Bold *P* values indicate statistical significance with *P* < 0.05

*BUN* blood urea nitrogen, *Cr* creatinine, *BMI* body mass index, *ASA* American Society of Anesthesiologists, *CHF* congestive heart failure, *COPD* chronic obstructive pulmonary disease

Compared to normal hydration, dehydration was significantly associated with a significantly greater likelihood of experiencing any complication (*P* = 0.004), nonhome discharge (*P* = 0.002), and LOS > 2 days (*P* < 0.001) (Table 2).

**Table 2:** Bivariate analysis of 30-day postoperative complications in patients with normal hydration and dehydration

Complications	Normal (BUN/Cr < 25)	Dehydration (BUN/Cr ≥ 25)	
	Number (%)	Number (%)	*P* value
Any complication	280 (24.4)	76 (33.8)	**0.004**
Sepsis	8 (0.7)	2 (0.9)	0.758
Septic shock	1 (0.1)	0 (0.0)	1.000
Pneumonia	2 (0.2)	0 (0.0)	0.997
Unplanned reintubation	3 (0.3)	0 (0.0)	0.999
UTI	10 (0.9)	1 (0.4)	0.519
Cardiac arrest or MI	3 (0.3)	2 (0.9)	0.179
Stroke	0 (0.0)	0 (0.0)	–
Blood transfusions	29 (2.5)	8 (3.6)	0.387
DVT	5 (0.4)	1 (0.4)	0.986
PE	6 (0.5)	1 (0.4)	0.880
On ventilator > 48 h	0 (0.0)	0 (0.0)	–
SSI	37 (3.2)	5 (2.2)	0.427
Wound dehiscence	4 (0.3)	0 (0.0)	1.000
Acute renal failure	0 (0.0)	2 (0.9)	0.997
*Clostridioides difficile* infection	1 (0.1)	0 (0.0)	1.000
Nonhome discharge	69 (6.0)	27 (12.0)	**0.002**
Readmission	60 (5.2)	10 (4.4)	0.624
Unplanned reoperation	40 (3.5)	10 (4.4)	0.485
Periprosthetic fracture	0 (0.0)	0 (0.0)	–
LOS > 2 days	173 (15.1)	58 (25.8)	** < 0.001**
Mortality	0 (0.0)	0 (0.0)	–

Bold *P* values indicate statistical significance with *P* < 0.05

*BUN* blood urea nitrogen, *UTI* urinary tract infection, *MI* myocardial infarction, *DVT* deep vein thrombosis, *PE* pulmonary embolism, *SSI* surgical site infection, *LOS* length of stay

After controlling for all significant associated patient demographic and comorbid factors, an adjusted multivariate regression analysis was conducted. Compared to normal hydration, dehydration was independently associated with a significantly greater likelihood of experiencing LOS > 2 days (OR 1.50, 95% CI 1.05–2.14; *P* = 0.028) (Table 3).

**Table 3:** Multivariate analysis of 30-day postoperative complications in patients with dehydration compared to normal hydration

Complications	Dehydration (BUN/Cr ≥ 25)
	OR, *P* value, (95% CI)
Any complication	1.27, 0.145, (0.92–1.75)
Nonhome discharge	1.39, 0.205, (0.84–2.29)
LOS > 2 days	1.50, **0.028**, (1.05–2.14)

Bold *P* values indicate statistical significance with *P* < 0.05

*BUN* blood urea nitrogen, *Cr* creatinine, *LOS* length of stay

## Discussion

This study investigated dehydration as a risk factor for 30-day postoperative complications following aseptic revision TSA. Analysis of a cohort of 1372 patients after exclusion from the NSQIP database who underwent revision TSA between the years of 2015 and 2022 revealed an association between dehydration and higher rates of 30-day postoperative complications compared to normal hydration. Relative to normal hydration, dehydration was found to be an independent predictor of a postoperative hospital stay exceeding 2 days following aseptic revision TSA.

Chronic suboptimal fluid intake, resulting in dehydration, has been linked to the development of many disorders including metabolic syndrome, type 2 diabetes mellitus, hypertension, coronary artery disease, and premature mortality [11]. Dehydration has also been linked to impaired vascular function and cardiovascular regulation, leading to reductions in exercise performance and orthostatic tolerance [12]. Dehydration is particularly prevalent in the elderly population and has been associated with adverse outcomes following surgery as well as during postoperative rehabilitation. One study reported that dehydration was prevalent in up to 60% of subjects aged 70 years and older [13]. Furthermore, dehydration in older adult surgical patients has been associated with an increased risk of mortality [14, 15]. Since revision TSA is commonly performed in older adults, investigating dehydration and its effects on this population is both relevant and warranted. In the present study, more than 84% of the patients included were ≥ 60 years. Notably, within the dehydration cohort over 90% of the patients were ≥ 60 years. This high prevalence of older patients underscores the importance of understanding how dehydration specifically impacts this age-group when undergoing revision TSA.

This study reports a 1.5-fold increased likelihood of dehydrated patients experiencing a length of stay that exceeds two days following aseptic revision TSA when compared to normal hydration. This further supports previous findings in shoulder procedures, as TSA has been independently associated with increased LOS [8]. Similarly, following total knee arthroplasty (TKA), dehydration was found to be associated with an increased risk of postoperative complications including nonhome discharge and extended LOS [6]. In addition, studies investigating the impact of dehydration on rehabilitation outcomes in older orthopedic patients reported increased adverse outcomes, including increased length of stay and mortality, associated with dehydration in the postoperative period [16, 17]. These findings underscore the need for optimal hydration management in orthopedic surgery and postoperative rehabilitation. Addressing dehydration in not only revision TSA, but other orthopedic procedures may significantly reduce complications and improve overall outcomes.

Extended LOS is a postoperative complication of significant concern in any procedure, but especially in revision TSA. A prolonged hospital stay increases the risk of hospital-acquired infections, which can lead to further complications and delay recovery [18, 19]. Infections can be particularly detrimental in older patients who have undergone multiple procedures, such as TSA and revision TSA. Additionally, these patients often have compromised immune systems and other comorbidities, making them more susceptible to severe complications from infections [19]. Additionally, extended LOS is often associated with higher healthcare costs, placing a greater financial burden on both patients and the healthcare system [20]. Furthermore, extended LOS can negatively impact patients’ mental health, leading to increased anxiety and other comorbid psychiatric problems [21]. Therefore, minimizing LOS is crucial to improving patient outcomes, reducing costs, and enhancing the overall health of the patient.

The present study has demonstrated that dehydration is independently associated with extended LOS, suggesting that preoperative patient stratification could be beneficial in preventing prolonged hospital stays in revision TSA. Preoperative laboratory values provide great utility in assessing modifiable risk factors and optimizing the patient prior to surgery. Specifically, preoperative dehydration is one of many modifiable risk factors and may be managed with appropriate fluid therapy [22]. This study provides evidence for the clinical utility of identifying patients with a BUN/Cr ratio ≥ 25 prior to revision TSA. By identifying and addressing dehydration through preoperative laboratory values, such as the BUN/Cr ratio, physicians can better manage modifiable risk factors and potentially reduce the incidence of extended LOS and associated complications. Implementing targeted fluid therapy, coupled with careful monitoring of at-risk patients, has the potential to significantly improve surgical outcomes and overall patient care. These interventions are both cost-effective and readily accessible, as intravenous fluid therapy is widely available across most healthcare settings. Future research should continue to investigate strategies for preoperative optimization, with a focus on interventions aimed at correcting preoperative dehydration, to enhance postoperative recovery and reduce hospital length of stay.

Limitations to this study include use of the NSQIP database, which contains information that is limited to a postoperative period of 30 days. Thus, we are unable to report on complications associated with preoperative dehydration occurring outside of this window. In addition, variables including surgical experience and the location at which procedures were performed are not available and may not be controlled for. Indications for revision TSA are not included in this database and therefore cases may not be stratified based on the risk associated with varying indications. Furthermore, causes other than dehydration that may lead to an elevated BUN/Cr ratio, including medication use and comorbid disorders such as cachexia, kidney disease, liver disease, and cardiac disease. In addition, dehydration may be defined using multiple metrics, and the BUN/Cr ratio is not universally agreed upon as the ideal metric for identifying dehydrated patients. It is important to note that preoperative laboratory studies included in the NSQIP database are typically obtained within 30 days prior to surgery. Accordingly, variability in the timing of laboratory collection relative to the operative date may have influenced the results of this study. Despite these limitations, the generalizability of our study is supported by an adequate sample size and the use of a comprehensive, multi-institutional database encompassing data from over 600 hospitals. These findings may aid in preoperative risk stratification by providing evidence that dehydration—defined by a BUN/Cr ratio ≥ 25—serves as an independent predictor of prolonged hospital stay following aseptic revision total shoulder arthroplasty.

## Conclusion

Our study identified dehydration, as defined by a BUN/Cr ratio of ≥ 25, as an independent predictor of a hospital stay exceeding two days following aseptic revision TSA. As the utilization of primary and revision TSA continues to rise, preoperative laboratory values, including the BUN to Cr ratio, may serve as a relatively inexpensive clinical tool in preoperative risk stratification. Recognizing the risks associated with preoperative dehydration may aid in identifying high-risk surgical candidates and optimizing their condition prior to aseptic revision total shoulder arthroplasty, potentially reducing the incidence of undesirable 30-day postoperative complications such as prolonged hospital stay.

## Data Availability

No datasets were generated or analyzed during the current study.
